# A Systemic Mapping Approach for Right and Left Parahisian Ventricular Arrhythmias Ablation

**DOI:** 10.3389/fcvm.2022.844320

**Published:** 2022-03-02

**Authors:** Ling-cong Kong, Tian Shuang, Zheng Li, Zhi-guo Zou, Wen-long Jiang, Jun Pu, Xin-hua Wang

**Affiliations:** Department of Cardiology, Ren Ji Hospital, Shanghai Jiao Tong University School of Medicine, Shanghai, China

**Keywords:** parahisian ventricular arrhythmias, systemic mapping approach, radiofrequency ablation, atrioventricular block, the septal leaflet of the tricuspid valve

## Abstract

**Background:**

Catheter ablation for parahisian ventricular arrhythmias (PHVA) is technically challenging and associated with increased risks of atrioventricular block (AVB). We developed a systemic mapping approach to improve the efficacy and safety of PHVA ablation.

**Methods:**

Forty-three patients (29 males; average age 65.8 ± 10.5 years) with PHVAs were enrolled. A systemic mapping approach comprising differential electrocardiogram, sequential mapping, and ablation beneath/above the septal leaflet of the tricuspid valve (SLTV) and at the neighboring/contralateral regions (the aortic root and sub-aortic valve region) was applied for PHVA. The effectiveness and safety of this approach was evaluated at 1 year's follow-up.

**Results:**

Sequential ablation beneath the SLTV (B-SLTV) succeeded in 24 (66.7 %) of 36 with right PHVA and ablation above the SLTV succeeded in 6 of the remaining 12 with failed B-SLTV ablation. Target-His bundle (HB) distance > 4.5 mm significantly predicted successful right PHVA ablation (OR 1.703; 95% CI 1.084–2.676, *P* = 0.02). “Seeming” right PHVA by electrocardiogram in 4 and apparent left PHVA in 3 was successfully ablated at the sub-aortic parahisian region. At 1 year's follow-up, 27 (75%) of 36 patients with right PHVA and 6 (85.7%) of 7 patients with left PHVA were free of PHVA recurrence off anti-arrhythmic drugs. The total success rate was 76.7% by using the systemic mapping approach for PHVA. One patient with A-SLTV ablation underwent pacemaker implantation due to complete AVB.

**Conclusions:**

The systemic mapping approach was effective and safe for treating PHVA. The target-HB distance was a significant predictor for right PHVA ablation.

## Highlights

- The systemic mapping approach comprising differential ECG, mapping and ablation beneath/above the SLTV and the neighboring/contralateral regions was effective and safe for treating right and left parahisian VAs.
- B-SLTV ablation succeeded in two thirds of patients with right parahisian VAs.
- Target-HB distance > 4.5 mm could predict successful ablation of right parahisian VAs.

## Background

Most idiopathic ventricular arrhythmias (VAs) [pre-mature ventricular contraction (PVC) and ventricular tachycardia (VT)] originate from the right ventricular outflow tract (RVOT), and can be eliminated by radiofrequency (RF) catheter ablation effectively (1, 2). Parahisian VA (PHVA), defined as the earliest activation exhibiting His potential or <1 cm away from the His bundle (HB) of the right or left ventricular side (3), are less commonly seen, with the prevalence of 3–9% in all idiopathic VAs (4, 5). Catheter ablation for PHVA is technically challenging because the septal leaflet of the tricuspid valve (SLTV) hampers catheter tip-tissue contacts by the “above SLTV (A-SLTV)” approach. In contrast to RVOT-VAs, PHVAs ablation is associated with compromised effectiveness and higher risks of conduction system injury (5–8).

A novel technical improvement is to curve the catheter tip reversely and place it beneath the SLTV (B-SLTV) to acquire good proximity to the basal interventricular septum (9, 10). The B-SLTV approach might be superior to the A-SLTV approach in terms of effectiveness and safety, and was recommended for PHVAs ablation in recent studies (10, 11); Nevertheless, catheter ablation might fail even by the B-SLTV approach in a considerable proportion of patients (11). In this scenario sequential mapping at the A-SLTV region, the neighboring left parahisian area or the aortic root might help to improve the outcomes (12). These findings reminded us to propose a systemic mapping approach for PHVAs ablation. This study was carried out to evaluate the effectiveness and safety of this approach in a consecutive cohort of patients with right and left PHVAs.

## Method

### Study Population

**Figure 1:**
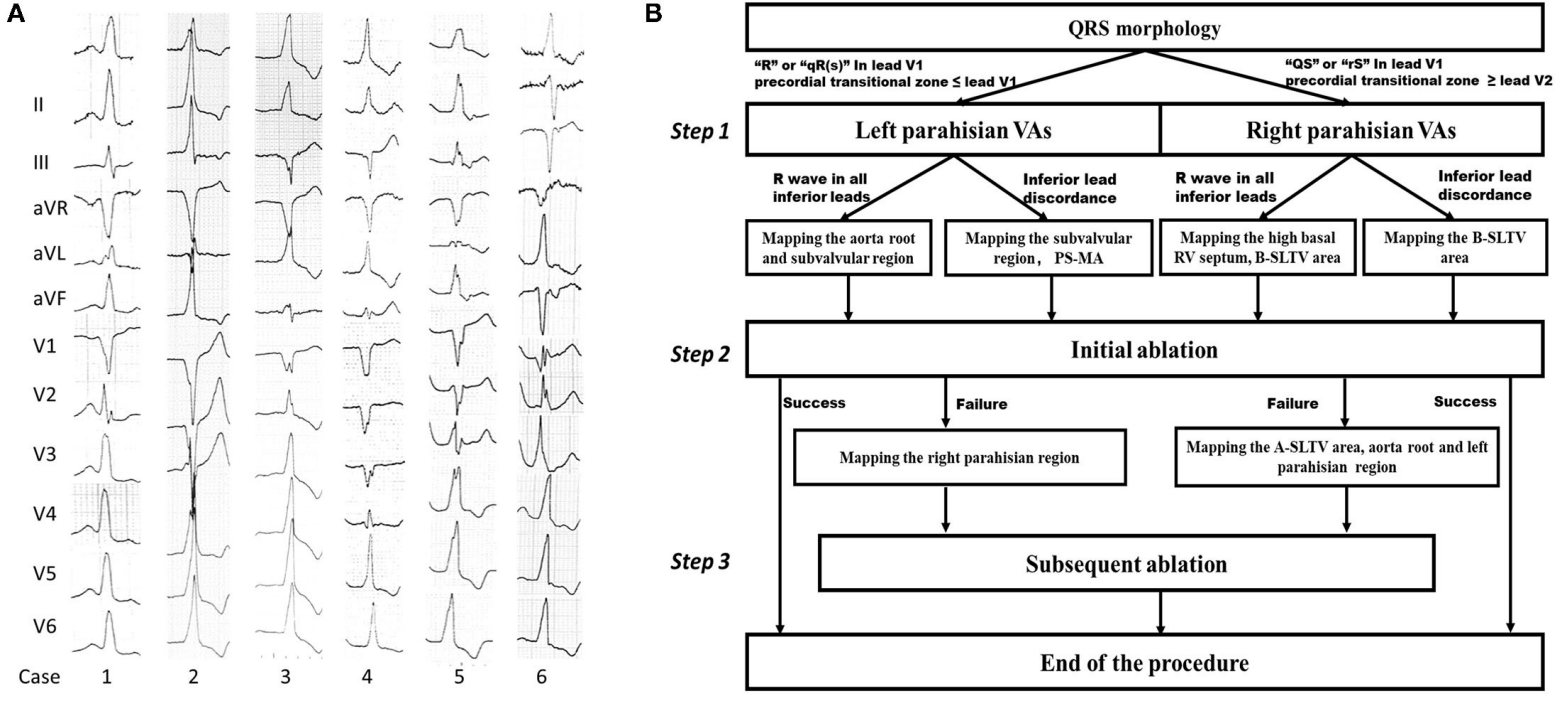
Examples of PHVA electrocardiograms and the flow chart of the systemic mapping approach. **(A)** Typical twelve-lead electrocardiographic manifestation of parahisian VAs. Right parahisian VAs were diagnosed in Case 1–5, and left parahisian VA in Case 6. Note there were positive “R” waves/or QRS discordance in inferior leads, a “QS” wave in lead V1, precordial transition in lead V2–V4 for right parahisian VAs, and a “qR” wave in lead V1 and precordial transition in lead V1–V2 for left parahisian VA. **(B)** The flow chart of the systemic mapping approach for right and left parahisian VA.

Forty-three patients (29 males; average age 65.8 ± 10.5 years) with PHVAs were enrolled to undergo catheter ablation from January 2017 to September 2020. PHVAs were diagnosed according to surface electrocardiograms (ECGs) (3–5), which typically manifested QRS discordance in inferior leads (lead II/III/aVF), “QS”/“Qr”/“qR”pattern in lead V1, and precordial transition earlier than lead V3/V4 (Figure 1A). Inclusion criteria were symptomatic monomorphic VAs; daily PVC burden > 10% or total PVC exceeding 10,000 per day or concomitant with VT of identical morphology; refractory or intolerance to at least one antiarrhythmic drug (AAD), including beta-blockers, Class Ic and Class III AADs. Exclusion criteria were ischemic heart disease; primary cardiomyopathy; prior cardiac surgery; congenital heart disease. All patients provided written informed consent for the ablation.

### The Protocol of Systemic Mapping Approach for PHVAs

The protocol of systemic mapping approach was shown in Figure 1B. Briefly, it could be described as three steps. Step 1: Pre-judgement of left or right PHVAs according to the QRS morphology in lead V1 and precordial transitional zone (13). The “R”/“qR(s)” pattern in V1 and precordial transitional zone ≤ V1 indicated a left PHVA. The “QS”/“rS” pattern in V1 and precordial transitional zone ≥ V2 indicated a right PHVA. Step 2: Initial ablation based on the results of parahisian mapping. For right PHVA, the B-SLTV approach was performed in all patients. For left PHVA, ablation was performed above or below the aortic valve based on the mapping results. Step 3: Subsequent mapping and ablation if the initial ablation failed, including A-SLTV ablation and anatomical ablation at the contralateral parahisian region and the adjacent structures.

### Electrophysiological Mapping

The procedures were performed in fasting state and under local analgesia. A decapolar catheter was positioned within coronary sinus (CS) *via* left femoral vein or left subclavian vein. A 3.5 mm saline-irrigated catheter with or without contact-force sensing (Thermocool Navistar or SmartTouch Thermocool Navistar, Biosense Webster, CA, USA) was used for geometry reconstruction, mapping and ablation. If spontaneous VAs was insufficient for mapping, intravenous infusion of isopreterenol (2–5 μg/min) was used to provoke the clinical VAs. The result of activation mapping was displayed on the geometry in color-coded manner. The site with the highest amplitude of His potential was tagged as the “HB.” Pace mapping was applied with the output just higher than the diastolic capture threshold. A matched pace mapping was defined as the morphology of paced QRS identical to that of the clinical VAs in at least 11 of 12 leads.

### EP Mapping at the Preferential Regions

**Figure 2:**
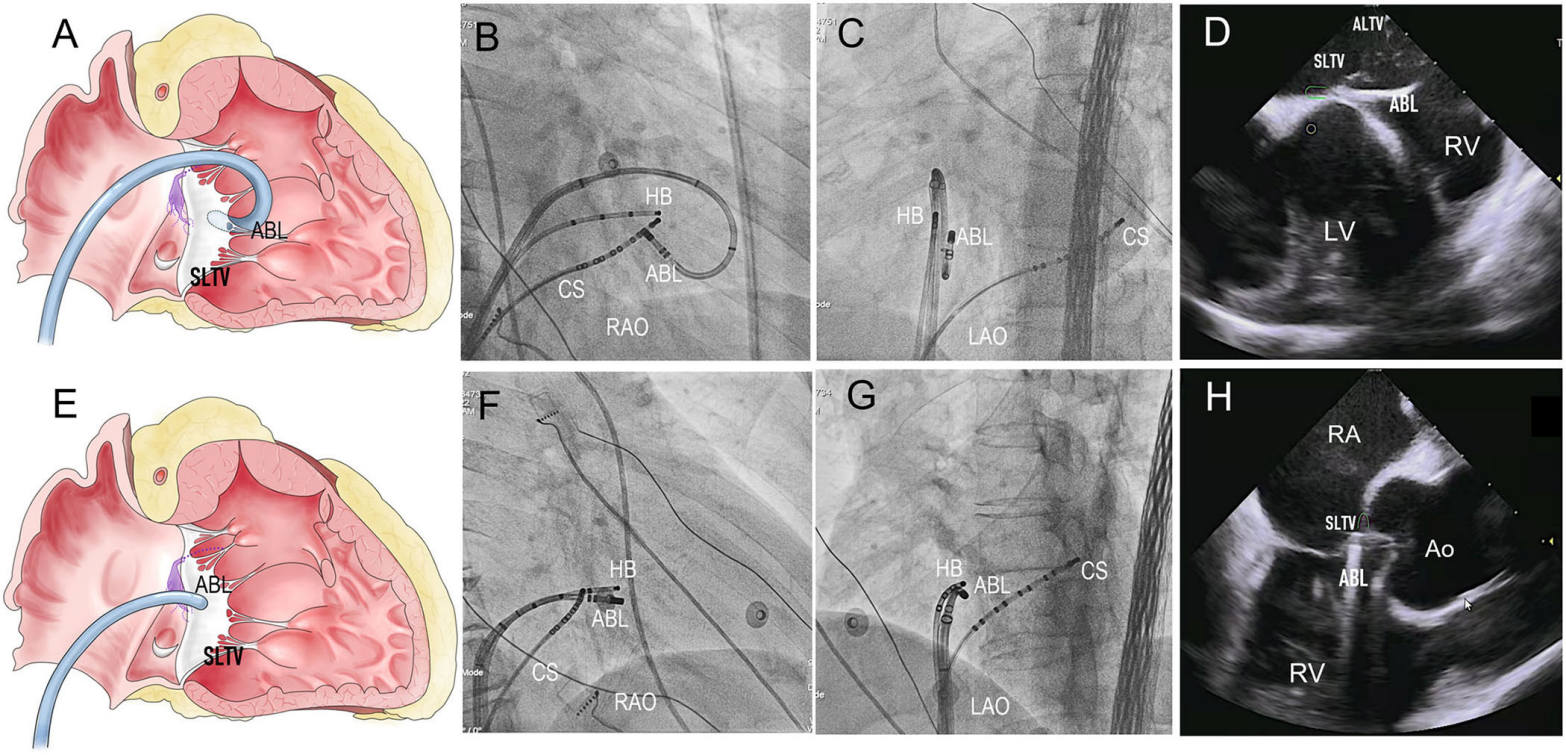
The Catheter placement in the B-SLTV and A-SLTV approach. **(A)** The schematic diagram illustrating the catheter tip was reversely curved beneath the SLTV (RAO view). **(B,C)** The fluoroscopic images showing the catheter was reversely curved beneath the SLTV through a long sheath (RAO and LAO view). **(D,H)** The ultrasonic images confirmed the catheter placement beneath the SLTV. **(E)** The schematic diagram showing the catheter tip was directly placed above the SLTV (RAO view). **(F,G)** The fluoroscopic images showing the catheter tip was placed directly above the SLTV (RAO and LAO view). HB, the His bundle; CS, coronary sinus; ABL, the ablation catheter; SLTV, the septal leaflet of the tricuspid valve; ALTV, the anterior leaflet of the tricuspid valve; RA, the right atrium; RV, the right ventricle; LV, left ventricle; RAO, right anterior oblique; LAO, left anterior oblique.

**Figure 3:**
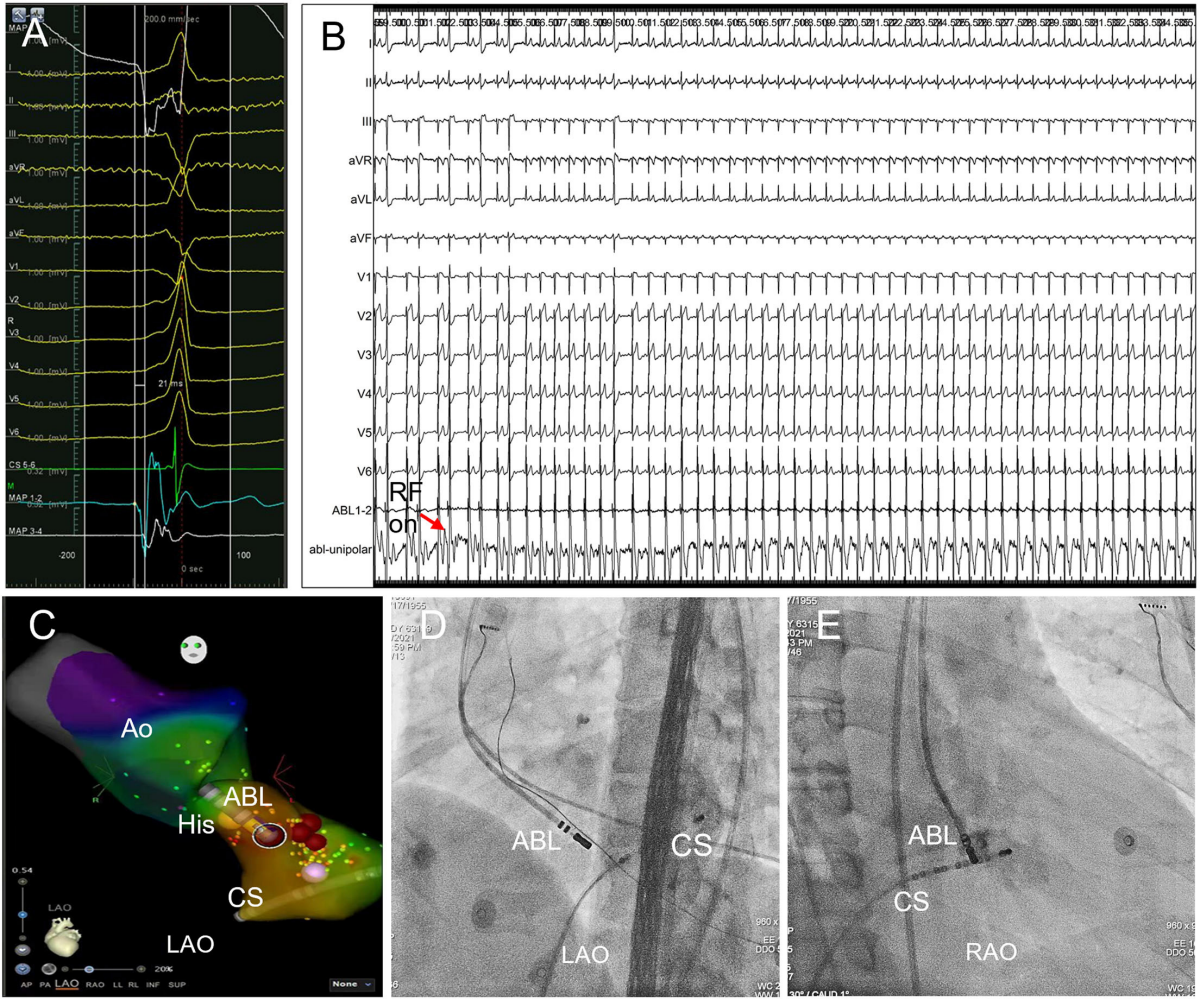
Trans-aortic retrograde ablation of left PHVA. **(A)** Tracings were unipolar MAP1, twelve-lead surface ECG, CS_5−6_, bipolar MAP_1−2_, MAP_3−4_. The local bipolar electrogram at the ablation target preceded the QRS onset by 21 ms with a sharp “QS” unipolar electrogram. **(B)** RF ablation 30W, 60s at the sub-aortic ablation target successfully eliminated the left PHVA without causing PR prolongation or AVB. **(C)** Activation mapping of the left PHVA region and the aortic root. The catheter tip was positioned at the ablation target ≈3 mm below the HB in the left ventricle. Ablation attempt (30W, 10 s) was not effective at other sites slightly away from the ablation target. Red dots represented ablation lesions. **(D,E)** The catheter placement at the left parahisian region on LAO and RAO fluoroscopic projection. Ao, the aorta; HB, the His bundle; CS, coronary sinus; ABL, the ablation catheter; RAO, right anterior oblique; LAO, left anterior oblique.

For right PHVAs, the B-SLTV region was preferentially mapped. To access to the subvalvular area, the ablation catheter was introduced to the right ventricle *via* a Swartz sheath (SR0 type, St. Jude Medical, MN, USA), then curved reversely and clockwise rotated the catheter and pulled it back toward the tricuspid annulus, to form catheter inversion configuration (Figures 2A–C). The position of the catheter tip was judged by fluoroscopy and confirmed by intracardiac echocardiography (ICE) imaging (CARTO Sound, Biosense Webster, CA, USA) (Figures 2D,H). For left PHVAs, the mapping catheter was advanced in the left ventricle *via* trans-aortic retrograde approach to map the left parahisian region and the septal region of mitral annulus (Figure 3).

**Figure 4:**
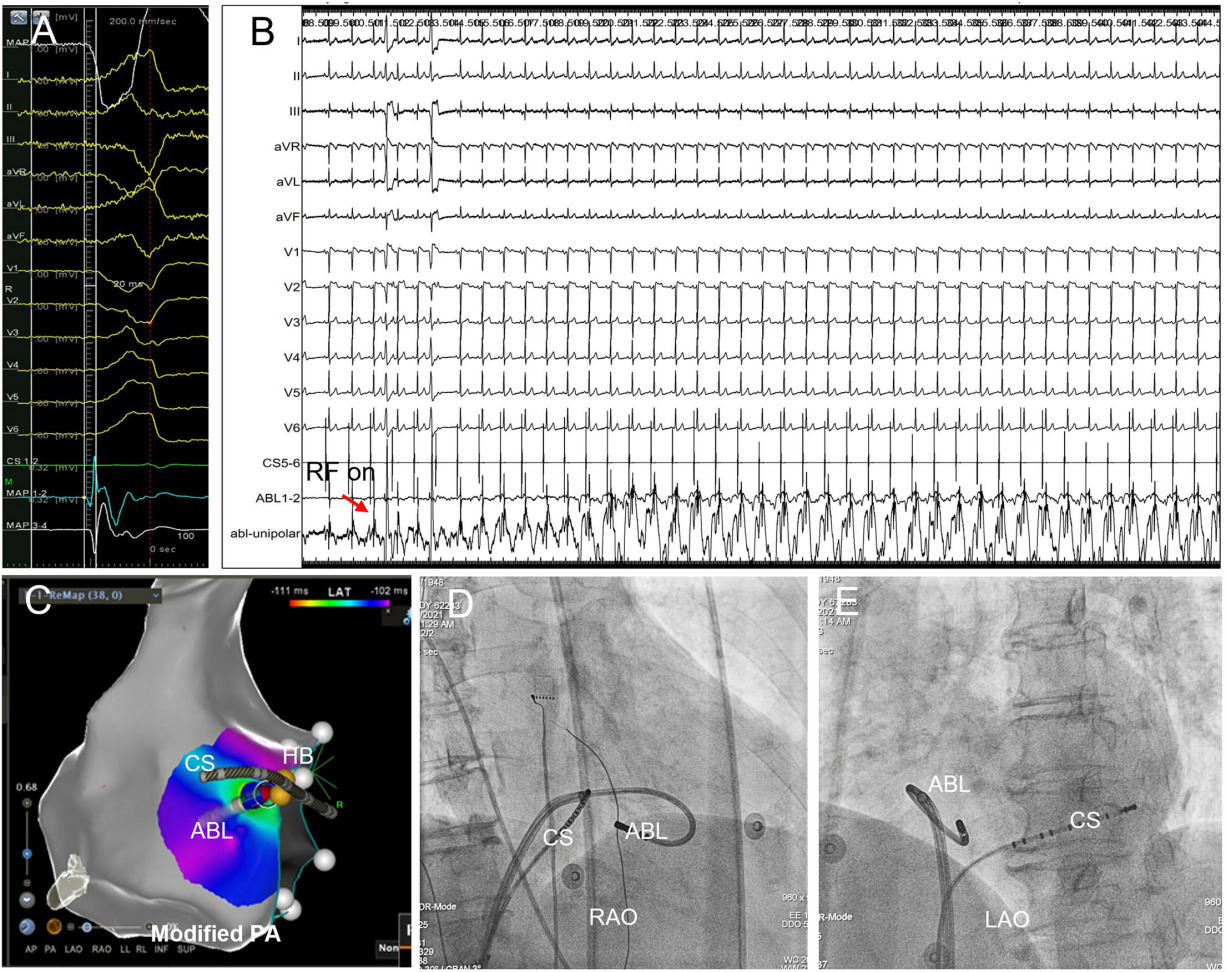
Successful B-SLTV ablation of right PHVA with the ablation target extremely close to the HB. **(A)** Tracings were unipolar MAP_1_, twelve-lead surface ECG, CS_1−2_, bipolar MAP_1−2_, MAP_3−4_. The local bipolar electrogram at the ablation target preceded the QRS onset by 20 ms with a sharp “QS” unipolar electrogram. **(B)** Saline-irrigated RF energy delivery 35W, 90s at this site successfully abolished the PVC, without junctional rhythm or AV conduction impairment. **(C)** The catheter tip was positioned beneath the SLTV ≈ 2 mm away from the HB. The blue dot represented the ablation lesion. **(D,E)** The B-SLTV placement of the ablation catheter was displayed on RAO and LAO projection. HB, the His bundle; CS, coronary sinus; ABL, the ablation catheter. PA, postero-anterior; RAO, right anterior oblique; LAO, left anterior oblique.

The earliest activation site was tagged on the geometry and served as the initial ablation target if it was >4 mm away from the HB, and if the HB-Right bundle branch (RBB) capture threshold by pacing at the earliest activation site was higher than that at the HB site (14). However, in case of the earliest activation <4 mm away from the HB, or if HB-RBB was captured with lower threshold at this site than the HB, attempt of ablation could be tried under close monitoring of junctional rhythm/AV conduction (Figure 4).

### Subsequent EP Mapping at Other Regions

If the attempt of B-SLTV ablation failed or was withdrawn due to safety concerns, the A-SLTV region (Figures 2E–G), contralateral parahisian regions and the neighboring structures (i.e., the coronary cusps at the aortic root) were mapped to search for an earlier activation site than the initial one, or the adjacent site that was just opposite to the initial lesion. They were served as a better target for subsequent ablation, or as a suitable target for anatomical ablation.

### RF Ablation Settings

Once the initial ablation target was determined, irrigated RF ablation was applied for lesion creation. Typically, RF energy delivery commenced at 20W (saline-irrigation speed 17 ml/min, contact force 8–20 g if applicable), and was gradually titrated to 35–45 W (saline-irrigation speed 20–25 ml/min, contact force 8–20 g if applicable) if no sign of AV conduction injury was observed (5–10). If the clinical VAs were suppressed within 15 s, RF energy delivery was prolonged for 90–120 s, otherwise RF ablation was suspended, and re-mapping was performed. RF ablation was terminated immediately in case of PR prolongation or rapid junctional rhythm, which foreboded impending AV block (AVB).

Procedural success was deemed if no clinical VAs recurred at the end of 30 min's observation period with the challenge of intravenous isoproterenol infusion (2–5 μg/min).

### Post-ablation Management and Follow-Up

Patients were discharged the next day after the procedure and were regularly followed up at the outpatient clinic for 1 year. All AADs were withdrawn if no symptomatic VAs were detected. Surface ECG and 24 h Holter monitoring were performed at 3, 6, and 12 months after the procedure, and were performed whenever the patient experienced symptoms suggesting a VA recurrence.

Clinical success was defined as absence of clinical PHVAs on surface ECGs and reduction of clinical VAs burden by more than 90% on 24 h Holter monitoring compared with the recording prior to the procedure at the end of 1 year's follow-up off AADs.

### Statistical Analysis

Continuous variables with normal distribution were expressed as mean ± standard deviation (SD) or as median and range otherwise. They were compared by Student's *t*-test if the variance was equal, or by Mann–Whitney *U*-test otherwise. Category variables were expressed as counts and percentage (%), and were compared with χ^2^ or Fisher's exact test. Multivariate binary logistic regression analysis was applied to detect the independent predictors for successful right PHVA ablation, which were described as odds ratio (OR) and 95% confidence interval (CI). A receiver operating characteristic (ROC) analysis was applied to determine the cut-off value for predicting successful right PHVA ablation. A two-tailed *P* < 0.05 was considered statistically significant. Statistical analysis was performed by IBM SPSS Statistics 19.0 for Windows (IBM Corporation, Somers, NY, USA).

## Results

The baseline demographic parameters in all patients and in those with right / left PHVAs were listed in Table 1, respectively. There were 18 patients with hypertension, 5 with type 2 diabetes mellitus, 2 with mild coronary artery atherosclerosis detected by computed tomographic angiography, and 3 with left ventricular ejection fraction 40–50%. Forty-two patients presented with isolated PVCs and 1 with concomitant non-sustained VT. An average of 1.3 ± 0.5 AADs were used per patient. The clinical VA was primarily diagnosed as left PHVA in 3 patients and as right PHVA in the remaining 40 patients based on the ECG. PHVA was successfully ablated from the left side in 4 of 40 patients with ECG-diagnosed right PHVA, and was re-grouped as left PHVA. Totally there were 36 with right PHVA and 7 with left-sided VA.

**Table 1:** Patients' baseline characteristics.

**Parameters**	**Total**	**Right parahisian VA**	**Left parahisian VA**
Cases (*N*)	43	36	7
Age (years)	65.8 ± 10.5	65.7 ± 11.2	66.4 ± 5.1
Male, *n* (%)	29 (67.4)	23 (63.9)	6 (85.7)
24-h PVC count	17,603 ± 7,072	16,878 ± 7,028	21,331 ± 6,528
History of PVC (months)	5.0 (4.0, 8.0)	5.0 (4.0, 7.8)	6.0 (3.0, 24.0)
**Comorbidities**
Hypertension, *n* (%)	18 (41.8)	15 (41.6)	3(42.9)
Diabetes mellitus, *n* (%)	5 (11.6)	4 (11.1)	1(14.3)
Mild coronary	2 (4.7)	2 (5.6)	0
atherosclerosis, *n* (%)			
LVEF 40–50%	3 (7.0)	2 (5.6)	1 (14.3)
**Presenting arrhythmia**
Isolated PVC, *n* (%)	42 (97.7)	35 (97.2)	7 (100)
PVC/NSVT, *n* (%)	1(2.3)	1 (2.8)	0
**AAD use**
Beta-Blocker, *n* (%)	20 (46.5)	17 (47.2)	3 (42.9)
Class Ic drug, *n* (%)	19 (44.2)	15 (41.7)	4 (57.1)
Sotalol, *n* (%)	10 (23.3)	8 (22.2)	2 (28.6)
Amiodarone, *n* (%)	6 (14.0)	6 (16.7)	0 (0)

*PVC, premature ventricular complex; NSVT, non-sustained ventricular tachycardia; LVEF, left ventricular ejection fraction; AAD, antiarrhythmic drug*.

### Right PHVA Ablation

**Figure 5:**
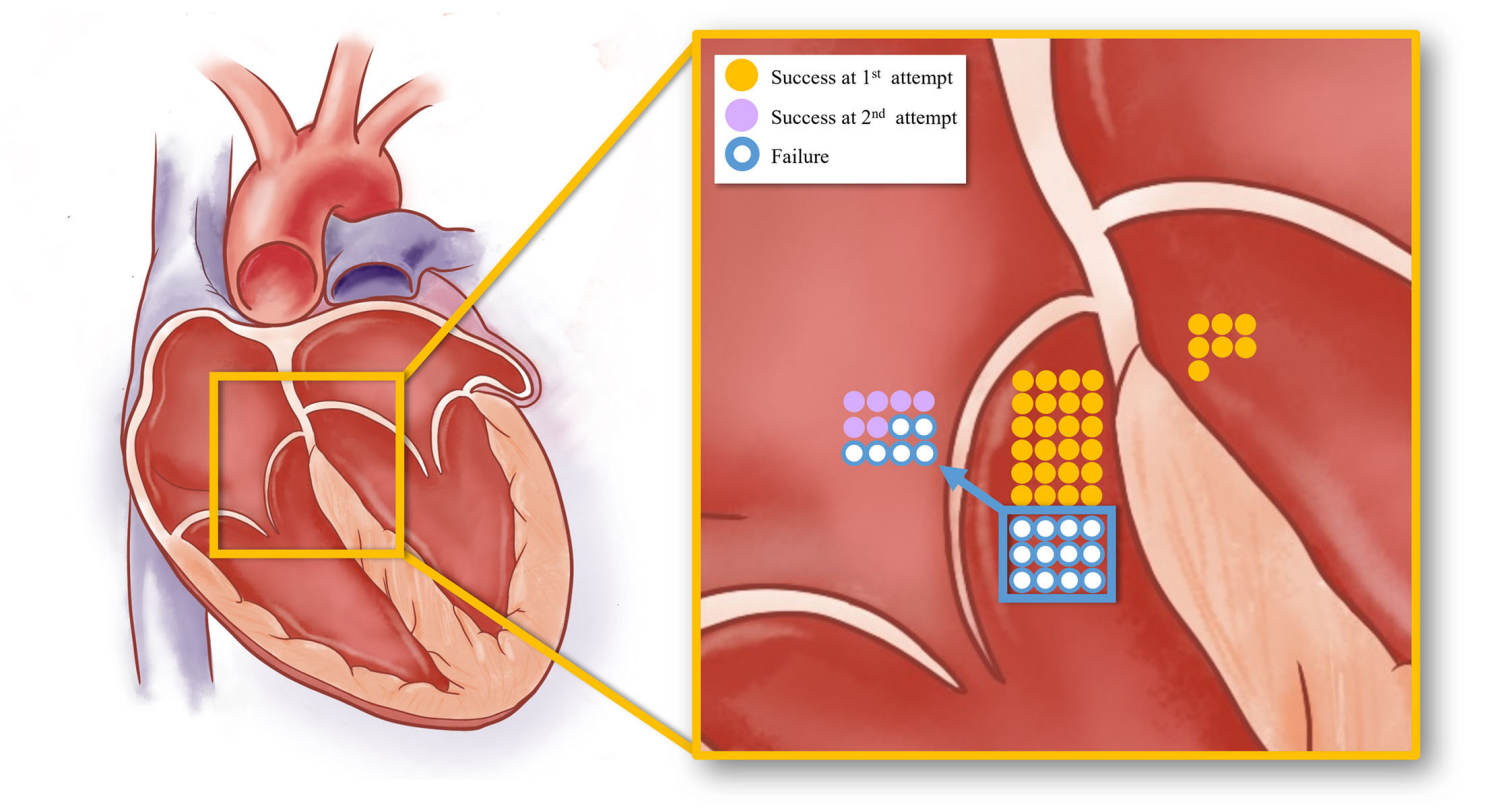
The schematic diagram showing the acute procedural results of right and left PHVA ablation. The yellow dots represented successful cases by the B-SLTV approach and those by trans-aortic retrograde ablation. The purple dots represented success cases by the adjunctive A-SLTV approach. White dots represented cases with ablation failure.

RF ablation by the B-SLTV approach succeeded in 24 (66.7%) of 36 patients with right PHVA, and failed in the remaining 12 patients (Figure 5). The electrocardiographic and procedural data were compared between successful and unsuccessful B-SLTV ablation in Table 2. There was significant greater target -HB distance and lower prevalence of junctional beats in patients with successful B-SLTV ablation than in those without. ECG morphologies, the local ventricular activation preceding QRS onset (V-QRS), proportion of small HB potential at the target, average RF power, RF duration, number of RF delivery and fluoroscopy time were comparable between successful and unsuccessful B-SLTV ablation.

**Table 2:** Comparison of right and left parahisian VA ablation.

	**Right parahisian** **VA ablation (*****n*** **=** **36)**			**Left parahisian VA ablation (*n* = 7)**
	**Total**	**Successful** **B-SLTV (*n* = 24)**	**Unsuccessful** **B-SLTV (*n* = 12)**	
**ECG morphology**
QS pattern in lead V1, *n* (%)	33 (91.7)^$^	23 (95.8)	10 (83.3)	2 (28.6)^$^
Precordial transition ≤ lead V2, *n* (%)	14 (38.9)^#^	8 (33.3)	6 (50.0)	6 (85.7)^#^
Inferior lead negative/discordance, *n* (%)	27 (75.0)	18 (75.0)	9 (75.0)	7 (100.0)
**Procedural parameters**
V-QRS, ms	24.8 ± 7.4	25.1 ± 7.1	24.1 ± 8.4	24.1 ± 5.7
Target-HB distance, mm	5.1 ± 2.3^‡^	5.8 ± 2.3^*^	3.7 ± 1.7^*^	7.4 ± 1.8^‡^
Small HBP at the target, *n* (%)	11(30.6)	5 (20.8)	6 (50)	2 (28.6)
Junctional beats, *n* (%)	10 (27.8)	2 (8.3)^*^	8 (66.7)^*^	2 (28.6)
RF power (W)	35.5 ± 3.0	35.1 ± 1.9	36.3 ± 4.3	36.9 ± 3.1
Number of RF delivery	3.6 ± 1.3	3.3 ± 1.2	4.0 ± 1.3	4.0 ± 1.2
RF duration (s)	150.6 ± 35.9	149.2 ± 37.2	153.4 ± 34.5	165.7 ± 41.5
Fluoroscopy time (s)	519.5 ± 352.7	441.6 ± 270.1	675.5 ± 451.1	346.1 ± 108.3

$ *P = 0.001;*

# *P = 0.04;*

‡ *P = 0.02 for comparison between right PHRA and left PHRA*.

* *P < 0.05 for comparison between successful B-SLTV and unsuccessful B-SLTV group*.

*RF, radiofrequency; HBP, the His bundle potential*.

Among the electrocardiographic and procedural parameters, target-HB distance was the only factor that could predict successful B-SLTV ablation for right PHVA (OR 1.703; 95% CI 1.084–2.676, *P* = 0.02). ROC analysis showed target-HB distance cutoff value was 4.5 mm (sensitivity 66.7% and specificity 83.3%; Area under the curve = 0.773, 95% CI 0.614–0.931, *P* = 0.01). There was no significant difference in RF power and duration between cases with target-HB distance > 4.5 mm and those with target-HB distance ≤ 4.5 mm (35.0 ± 2.3 vs. 36.0 ± 3.4 W, *P* = 0.34; 155.5 ± 38.1 vs. 146.3 ± 34.2 s, *P* = 0.76, respectively).

Subsequent ablation in 12 patients with unsuccessful B-SLTV ablation was listed in detail in Supplementary Table 1. A-SLTV ablation successfully abolished the clinical VA in 6 of them. Totally procedural success was achieved in 30 (83.3%) of 36 patients with right PHVA. The remaining 6 patients experienced ablation failure even after thorough mapping and ablation at the neighboring structures (RVOT and three coronary cusps at the aortic root) and the left- parahisian region.

### Left PHVA Ablation

Sub-aortic valve ablation was successful in all seven patients with left PHVA. In each of 4 patients with failed right parahisian ablation, the V-QRS time at the left-sided target was greater than that at the right-sided target (27 vs. 24, 31 vs. 29, 16 vs. 14, 33 vs. 31 ms, respectively). Compared with right PHVA, left PHVA exhibited higher proportion of precordial transition ≤lead V2 and lower proportion of QS shape in lead V1 on ECG recordings (Table 2). The procedural parameters were comparable except that the target-HB distance was greater in left sided group than that in right-sided group.

### Complications

Mechanical injury caused right bundle branch block in 6 patients during catheter manipulation in the RV, which recovered in all of them within 24 h post-ablation. Transient high degree AVB occurred during A-SLTV ablation 4 mm below the HB in 1 patient, and resolved completely after immediate ablation suspension. However, this patient underwent pacemaker implantation due to complete AV block one- month post-ablation.

### Follow-Up Results at 1 Year

At the end of 12 months' follow-up, clinical success was achieved in 27 (75%) of 36 patients with right PHVA and in 6 (85.7%) of 7 patients with left PHVA. In this PHVA cohort, totally the clinical success was achieved in 33 (76.7%) of 43 patients off AADs by using this systemic mapping approach.

## Discussion

### Major Findings

In this retrospective study we firstly proposed and evaluated a novel systemic mapping approach for PHVA ablation, which was a combination of ablation at the B-SLTV/A-SLTV region, the neighboring structures and the contralateral parahisian regions. Secondly the target-HB distance was the only predictor for right PHVA ablation among all the procedural parameters and this value exceeding 4.5 mm predicted successful ablation. This informative result might provide us a useful tool to estimate the effectiveness in advance and avoid excessive risks of AVB during the procedure.

### Challenges and Obstacles for PHVAs Ablation

In one anatomical study it was found that the HB ran superficially without a layer of myocardial coat (the “naked” HB) in one fifth of the subjects (15). In another study it was found that the distal compact atrioventricular node (AVN) and the proximal HB had no surrounding myocardial layer (16). In this scenario RF ablation in close proximity to the AVN-HB was associated with increased risk of AVB. This severe complication became a major challenge for PHVA ablation. The major anatomical obstacle that was faced during right PHVA ablation was the SLTV. The “shield effect” of the SLTV prevented direct energy delivery to the basal myocardium. Furthermore, the movement of the SLTV might cause displacement of catheter tip, leading to insufficient lesion creation.

### Technical Innovations in PHVA Ablation

In the early days, the conventional A-SLTV approach was initially used for right PHVA ablation in several small-volume studies (6–8), with modest effectiveness and safety results. Later on, the application of cryo-energy failed to demonstrate better efficacy and safety in right PHVA ablation, compared with RF energy (17). In that study, cryoablation succeeded in only 4 of 10 patients with PHVA with the cost of pacemaker implantation in 1 patient complicated with three-degree AVB. The potential advantages of cryo- over RF energy comprised reversible cryo-mapping with a temperature of −30°C, reduced risk of damage to the surrounding tissue and better catheter stability. However, the size of ice ball was bigger than that of the catheter tip during cryoablation at a temperature of −70°C, which might reduce the distance threshold to avoid AVB. Furthermore, the A-SLTV rather than the B-SLTV approach was applied in that study. The latter might be more useful to improve the effectiveness as well as safety for PHVA ablation, which would be discussed below.

In recently years, the novel B-SLTV ablation (also known as “catheter inversion” technique, “reversed C-curve technique,” etc.) began to be applied for PHVA ablation, with a high success rate of 88% reported in a latest cohort study of 28 cases (11). The advantages of the B-SLTV approach over the conventional A-SLTV approach lay in two aspects: one was better catheter tip-tissue contact and more efficient energy delivery; the other was less risk of AVB, which was demonstrated in an animal study. It that study, the B-SLTV ablation carried less risk of AVB in experimental dogs compared with the A-SLTV ablation (18).

Judgement of B-SLTV placement of the catheter tip was vital for the B-SLTV approach. Although the fluoroscopic catheter inversion configuration indicated correct placement, it was doubtful that the catheter tip was at the right place in some cases. At this time the B-SLTV positioning could be judged by the fluoroscopic sign of catheter “fixation” while pulling it back, and could be further confirmed by ICE imaging (shown in Figures 2D,H). Even if the catheter tip was placed at the B-SLTV area, a contact-force sensing catheter was preferable and sometimes clockwise rotation of the catheter was needed to ensure appropriate catheter tip-tissue contact.

### Rationale for a Systemic Mapping Approach for Right PHVA Ablation

Despite the important advantages, the B-SLTV approach was not the end of story in the course of right PHVA ablation. It might not be able to abolish left parahisian foci, intramural foci, or foci extremely close to the AVN-HB. In our cohort, the procedural success could only be achieved in 66.7% of the patients by the B-SLTV approach. The “seemingly” right PHRA turned out to be left PHVA in a small proportion of the patients. Similar to RVOT VA ablation, VA with a left-sided origin might breakout from a right-sided exit through a preferential conduction pathway (19, 20), exhibiting the ECG of a right-sided VA. In our study the earliest local activation time at the left side preceding the one at the right side supported the existence of preferential conduction. Apparently, the B-SLTV approach was not effective for left PHVAs and left parahisian ablation was necessitated. Furthermore, to abolish intramural foci, RF ablation in the contralateral region might be necessary, rather than repeated B-SLTV ablation. Moreover, to ablate the ectopic focus extremely close to the AVN-HB, the B-SLTV approach was not always effective or safe and the A-SLTV approach/anatomical ablation at the neighboring structures might be effective (11, 12). The adjunctive A-SLTV approach succeeded in 6 of 12 cases who experienced B-SLTV ablation failure in our study. Among 6 cases with adjunctive A-SLTV ablation success, the target-HB distance was only 2 mm in 2 cases. This result indicated that different catheter tip orientation and trivial adjustment of the target site might help to improve the ablation results on certain occasions, though the risk of AVB should be carefully balanced.

### Predictors for Successful PHVA Ablation

There were no electrocardiographic predictors (such as QRS shape in lead V1 and the inferior leads, precordial transition, etc.) identified for successful PHVA ablation. However, among all the procedural parameters, we found only the target-HB distance > 4.5 mm could predict successful right PHVA ablation. We believed this finding might serve as a useful tool to estimate the effectiveness ahead of ablation and avoid excessive risks of AVB if the target-HB distance was markedly shorter than 4.5 mm.

### Study Limitations

Several major limitations existed in our study. Firstly, this study was a retrospective, single-arm cohort study and included limited patients. The effectiveness and safety of the systemic mapping approach should be tested in a prospective, controlled, and large-scale clinical trials. However, given the fairly low prevalence of PHVA, all the published literature were mainly case reports or small case series reports, lacking randomized controlled studies. Secondly, it was possible that PHVA abolished at left side after failed at right side originated from an intramural focus rather than a true left side focus. However, as was the case in RVOT VA, the existence of preferential conduction might increase the difficulties in identifying left or right out-flow tract VAs. The earliest local activation time at the left-sided target preceding the one at the right-sided target supported the existence of preferential conduction in our study. There might be no gold standard for defining right, intramural and left foci, and the response to ablation might be a practical one. Successful ablation at the contralateral parahisian region or the adjacent structures justified the application of s systemic mapping approach in complex PHVA ablation.

## Conclusions

The systemic mapping approach was effective and safe for treating right and left PHVAs. The ablation target-HB distance was a significant predictor for right PHVA ablation.

## Data Availability Statement

The raw data supporting the conclusions of this article will be made available by the authors, without undue reservation.

## Author Contributions

XW conceptualized the systemic mapping approach for PHVA ablation. LK, XW, and TS drafted the manuscript. LK drew the figures and illustrations. ZL were involved in clinical data collection. ZZ and WJ were involved in data processing. JP revised the manuscript. All authors have read and approved the final manuscript.

## Funding

This study was supported by funds from Shanghai Natural Science Foundation Project (Grant No. 18ZR1423400), National Science Fund for Distinguished Young Scholars (81625002), and Shanghai Outstanding Academic Leaders Program (18XD1402400). These funds were not used in study designing or data collection/processing.

## Conflict of Interest

The authors declare that the research was conducted in the absence of any commercial or financial relationships that could be construed as a potential conflict of interest.

## Publisher's Note

All claims expressed in this article are solely those of the authors and do not necessarily represent those of their affiliated organizations, or those of the publisher, the editors and the reviewers. Any product that may be evaluated in this article, or claim that may be made by its manufacturer, is not guaranteed or endorsed by the publisher.

## Abbreviations

PHVA: parahisian ventricular arrhythmias
PVC: premature ventricular contraction
VA: ventricular arrhythmia
VT: ventricular tachycardia
RF: radiofrequency
HB: the His bundle
RVOT: the right ventricular outflow tract
AVB: atrioventricular block
SLTV: the septal leaflet of the tricuspid valve
AAD: antiarrhythmic drug.
