# Supplementary material for: A Systemic Mapping Approach for Right and Left Parahisian Ventricular Arrhythmias Ablation
Source: Front Cardiovasc Med. 2022 Mar 2;9:844320. doi: 10.3389/fcvm.2022.844320 (PMC8924133; doi:10.3389/fcvm.2022.844320)
Supplement: Supplementary file 1 [file Table_1.docx]

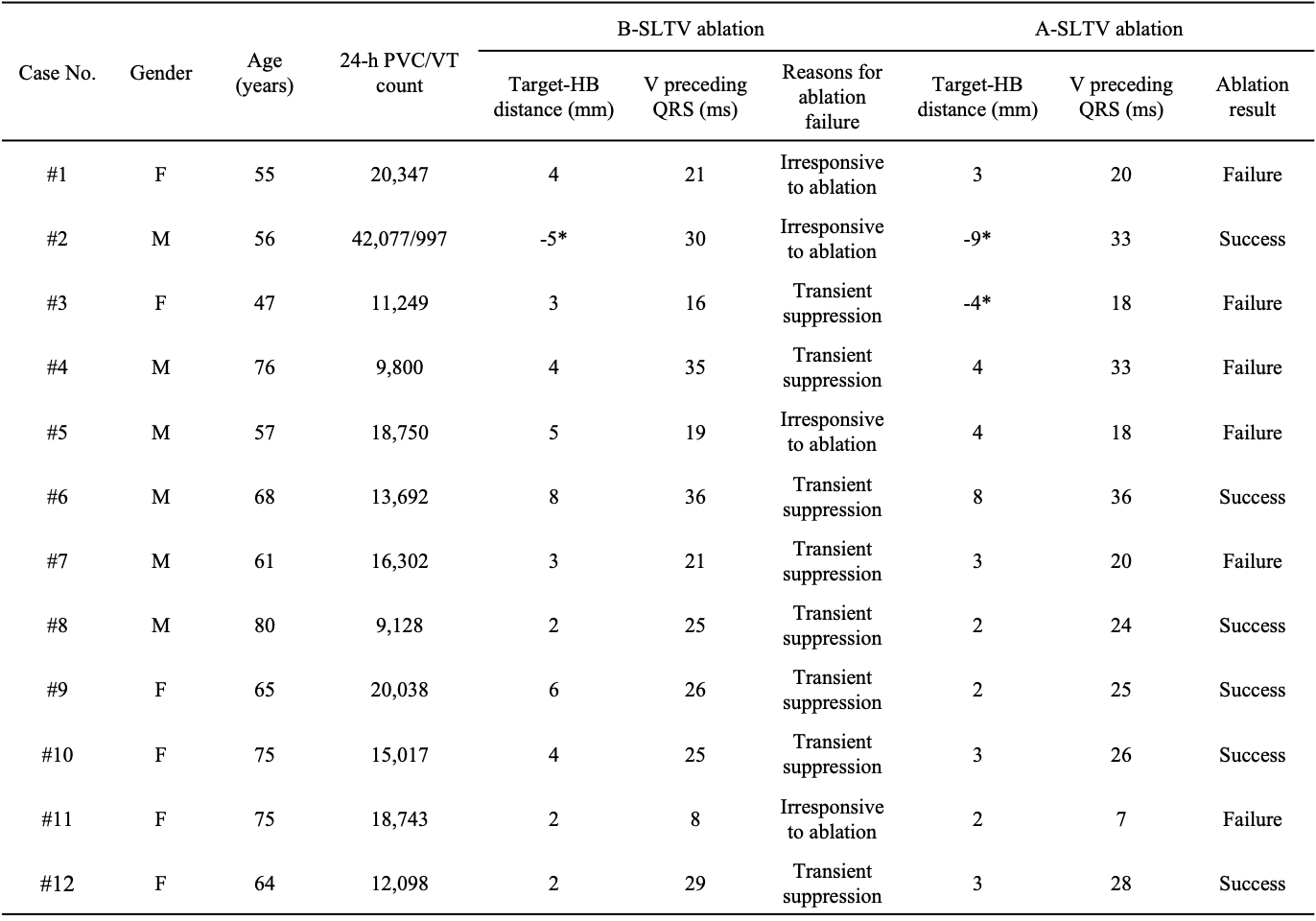


** Negative value represented the ablation target higher than the HB level*

**Supplemental Table 1**

**Results of A-SLTV ablation after failed B-ALTV ablation**
